# Whole brain radiotherapy versus stereotactic radiosurgery for 4–10 brain metastases: a phase III randomised multicentre trial

**DOI:** 10.1186/s12885-017-3494-z

**Published:** 2017-07-25

**Authors:** Jaap D. Zindler, Anna M. E. Bruynzeel, Daniëlle B. P. Eekers, Coen W. Hurkmans, Ans Swinnen, Philippe Lambin

**Affiliations:** 1 Department of Radiation Oncology (MAASTRO clinic), GROW School for Oncology and Developmental Biology, Maastricht University Medical Centre, dr. Tanslaan 12, 6229ET, Maastricht, the Netherlands; 2 Department of Radiation Oncology, VU University Medical Center, Boelelaan 1117, 1081 HV, Amsterdam, the Netherlands; 3 Department of Radiation Oncology, Catharina Hospital Eindhoven, Michelangelolaan 2, 5623 EJ, Eindhoven, the Netherlands; 4 MAASTRO clinic, P.O. Box 3035, 6202 NA Maastricht, The Netherlands

**Keywords:** Brain metastases, Stereotactic radiosurgery, Whole brain radiotherapy, Quality of life

## Abstract

**Background:**

Maintenance of quality of life is the primary goal during treatment of brain metastases (BM). This is a protocol of an ongoing phase III randomised multicentre study. This study aims to determine the exact additional palliative value of stereotactic radiosurgery (SRS) over whole brain radiotherapy (WBRT) in patients with 4–10 BM.

**Methods:**

The study will include patients with 4–10 BM from solid primary tumours diagnosed on a high-resolution contrast-enhanced MRI scan with a maximum lesional diameter of 2.5 cm in any direction and a maximum cumulative lesional volume of 30 cm^3^. Patients will be randomised between WBRT in five fractions of 4 Gy to a total dose of 20 Gy (standard arm) and single dose SRS to the BMs (study arm) in the range of 15–24 Gy. The largest BM or a localisation in the brainstem will determine the prescribed SRS dose. The primary endpoint is difference in quality of life (EQ5D EUROQOL score) at 3 months after radiotherapy with regard to baseline. Secondary endpoints are difference in quality of life (EQ5D EUROQOL questionnaire) at 6, 9 and 12 months after radiotherapy with regard to baseline. Other secondary endpoints are at 3, 6, 9 and 12 months after radiotherapy survival, Karnofsky ≥ 70, WHO performance status, steroid use (mg), toxicity according to CTCAE V4.0 including hair loss, fatigue, brain salvage during follow-up, type of salvage, time to salvage after randomisation and Barthel index. Facultative secondary endpoints are neurocognition with the Hopkins verbal learning test revised, quality of life EORTC QLQ-C30, quality of life EORTC BN20 brain module and fatigue scale EORTC QLQ-FA13.

**Discussion:**

Worldwide, most patients with more than 4 BM will be treated with WBRT. Considering the potential advantages of SRS over WBRT, i.e. limiting radiation doses to uninvolved brain and a high rate of local tumour control by just a single treatment with fewer side effects, such as hair loss and fatigue, compared to WBRT, SRS might be a suitable alternative for patients with 4–10 BM.

**Trial registration:**

Trial registration number: NCT02353000, trial registration date 15^th^ January 2015, open for accrual 1^st^ July 2016, nine patients were enrolled in this trial on 14^th^ April 2017.

## Background

**Fig. 1:**
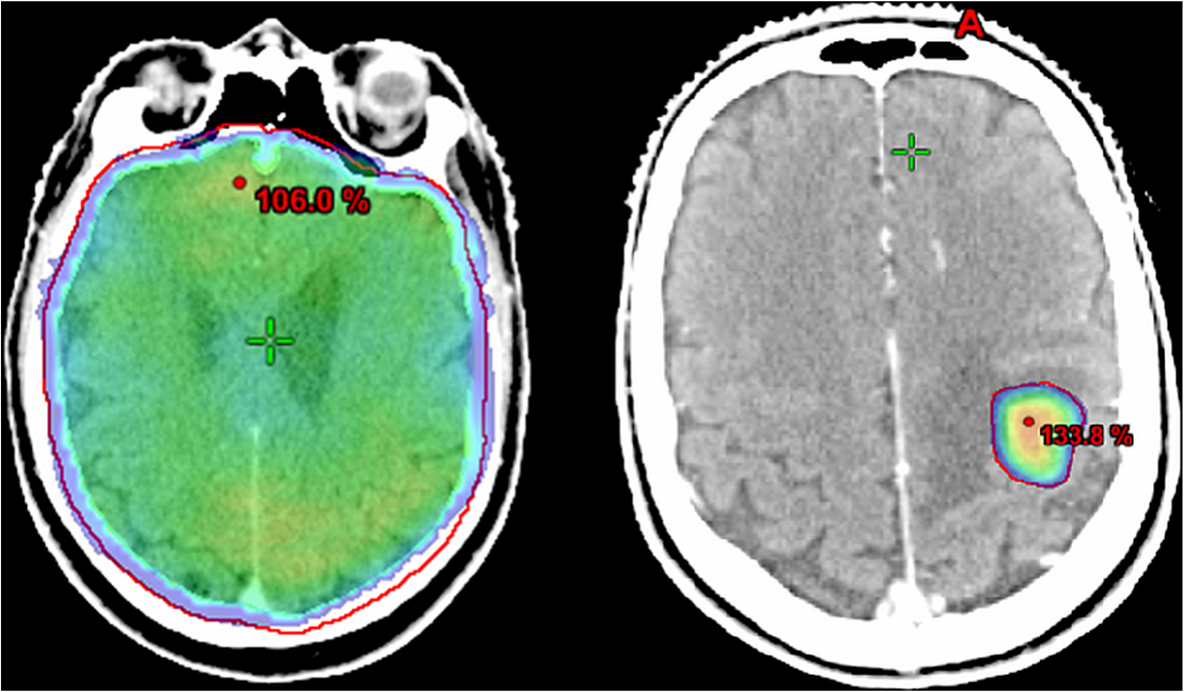
Dose distribution difference between WBRT (*left*) and SRS (*right*). A typical dose distribution on a planning-CT of WBRT on the left side and SRS on the right side. With WBRT, the healthy brain tissue receives the same low palliative radiation dose (non-ablative). With SRS, only the metastatic tissue receives a high ablative dose

In this randomised study, in patients with 4–10 brain metastases (BM), the standard treatment of whole brain radiotherapy (WBRT) is compared to stereotactic radiosurgery (SRS) for all lesions with the primary endpoint of quality of life (QOL) at 3 months after radiotherapy. We hypothesise that SRS provides better QOL than WBRT because of better local tumour control and avoidance of potential side effects of WBRT. Brain metastases are an important cause of morbidity and mortality in patients with metastasised cancer, and therefore, optimal tumour control is essential. Dutch guideline recommends SRS for patients with 1–3 BM and WBRT for patients with 4 or more BM. WBRT has side effects such as hair loss, fatigue and cognitive dysfunction, which may result in decreased QOL that is undesirable in a palliative setting. [1] There are important advantages of SRS over WBRT, i.e., limiting radiation doses to the uninvolved brain and a high rate of local tumour control by just a single treatment compared to WBRT, in which a relatively low palliative radiation dose is delivered to both the brain and the BM (Fig. 1). SRS is widely available in most Dutch radiotherapy centers. Because of recent technical advances, SRS can be delivered in relatively short treatment time in 10–45 min in patients with multiple BM. With SRS, there is a relatively low risk (±5%) of symptomatic radionecrosis: damage of the surrounding brain tissue, which may occur several months after treatment. Radionecrosis may cause neurologic symptoms, often temporary, which are treated with dexamethasone. Moreover, the clinical value of WBRT over the best supportive care is controversial. A recent interim analysis of the QUARTZ study showed equal QOL and survival for patients treated with WBRT versus treatment with steroids alone. [2] A recent (non-randomised) study in a large cohort of patients with BM showed that after SRS, survival of patients with 5–10 BM was comparable to that of patients treated with 2–4 BM. [3] Thus far, WBRT has never been compared directly with SRS in patients with 4–10 BM, and therefore a randomised trial is needed. In the United States, the NAGKC 12–01 (NCT01731704) was initiated in patients with 5 or more brain metastases in which SRS was directly compared with WBRT. However, this study was closed prior to enrolment of patients because of insufficient staff. Many systemic therapies do not have satisfactory tumour control of BM because of poor passage of the blood brain barrier. In the future, SRS may be the optimal treatment choice to control BM in patients with multiple brain metastases to maintain long-term QOL, whereas new innovative systemic therapies may control extracranial disease.

## Methods/design

### Design

The study is a randomised phase III study with two study arms. The standard arm is WBRT and the experimental arm is SRS. We hypothesise that SRS provides better QOL than WBRT because of better local tumour control and avoidance of potential side effects of WBRT.

### Objectives and endpoints

The primary objective is to determine whether QOL is better preserved after SRS than after WBRT in patients with 4–10 BM. The primary endpoint is difference in QOL (EQ5D EUROQOL score) at 3 months after radiotherapy with regard to baseline. The secondary objective is to determine whether SRS provides better survival and less toxicity than WBRT. [1, 2] Secondary endpoints are difference in QOL (EQ5D EUROQOL questionnaire) at 6, 9 and 12 months after radiotherapy with regard to baseline, survival at 3, 6, 9 and 12 months after radiotherapy, Karnofsky ≥ 70, WHO performance status, steroid use (mg), toxicity according CTCAE V4.0 including hair loss, fatigue, neurocognition and brain salvage during follow-up, type of salvage, time to salvage after randomisation and Barthel index. Facultative secondary endpoints are neurocognition with the Hopkins verbal learning test, quality of life EORTC QLQ-C30, quality of life EORTC BN20 brain module and fatigue scale EORTC QLQ-FA13.

### Study population

The study will include patients with 4–10 BM from solid tumours diagnosed on a high resolution contrast-enhanced MRI scan referred for radiotherapy, with a maximum lesional diameter of 2.5 cm. Before randomisation, a new neuronavigation MRI (T1 gadolinium) is made for the definitive evaluation of the inclusion and exclusion criteria. The inclusion criteria are age ≥ 18; minimum of 4 BM up to a maximum of 10 BM on diagnostic MRI scan; maximum diameter of single gross tumour volume (GTV) 2.5 cm; maximum cumulative GTV of 30 cm^3^; Karnofsky performance status ≥70; any solid primary tumour, and patients’ ability to provide written informed consent. Small cell lung carcinoma, germinoma and lymphoma are excluded. Other exclusion criteria are a contraindication for MRI, prior treatment for BM (i.e. surgery, SRS or WBRT), concurrent use of systemic therapy (systemic therapies should be stopped at least 1 week prior until 1 week after the radiotherapy), maximum cumulative GTV of more than 30 cm^3^ on planning-MRI, more than 10 BM on planning-MRI, leptomeningeal disease and brainstem metastasis with a PTV of more than 20 cm^3^. If a patient is not eligible based on the inclusion or exclusion criteria of this study based on the planning-MRI results prior to randomisation (e.g. >10 BM, GTV diameter > 2.5 cm, cumulative GTV > 30 cm^3^), these non-eligible patients will be replaced by a new patient. These patients are not included in the statistical analysis of the trial.

### Study procedures WBRT

On a gadolinium contrast-enhanced (single – triple dose Gd is allowed) MRI (1.0 T–3 T) with a maximal slice thickness of 1.5 mm, the definitive number of BM and the definitive maximum lesion diameter in any direction of the largest BM are determined. Patients randomised for WBRT will be treated with five fractions of 4 Gy up to a total dose of 20 Gy delivered in five consecutive working days. Dose prescription is according to ICRU 50 criteria. [4] The brain is contoured as a clinical target volume (CTV) until the foramen magnum. The CTV is equal to the PTV. To determine the size of the GTVs of the metastases, all BMs and lenses are contoured. Patients are positioned with a mask. The use of a planning CT is mandatory with slice thickness of ≤3 mm. The use of a contrast medium is not obliged. The planning-MRI is co-registered for contouring of the BM. The daily prescription dose will be 4 Gy prescribed at the ICRU reference point, and the 95% isodose must encompass 99% of the planning target volume (PTV); the maximum dose to the PTV should not exceed 107% of the prescribed dose. Generally, two opposed lateral fields are used with shielding of lenses and the pharyngeal space. All techniques that result in the dose requirements being met are allowed.

### Study procedures of SRS

Only single fraction treatments are allowed within this protocol. For any given patient, all brain metastases will be treated with the same dose, which will be determined by the PTV of the largest BM or brainstem location in the range of 15–24 Gy. (Table 1) The dose gradient outside the PTV will be as steep as possible to spare healthy brain tissue. Within the PTV, there will be considerable dose inhomogeneity, with a maximum allowed dose within the PTV of 140% of the prescribed dose. The GTV is defined by contouring the outer contrast-enhancing border of the BM on T1 gadolinium-weighted MRI images. BM are named GTVp1, GTVp2, GTVp3, from the cranial to the caudal side. Organs at risk (brainstem, optic nerves, chiasma, pituary gland, cochleae, and lenses) are contoured according to Scoccianti et al. [5]. The PTV is defined by a 0–2 mm isotropic expansion of the GTV, according to institutional standards for SRS. If a BM is within or adjacent to the brainstem, the PTV margin will be 0 mm. If in an institution, a smaller GTV to PTV margin is used when lesions are treated using multiple isocentres, then this technique is to be considered to reduce the V12_Gy_ of the largest BM if it would otherwise be more than 10 cm^3^.

**Table 1:** Risk adapted SRS dose prescription volume and location based

PTV of the largest brain metastasis	Doses in each PTV	BM in brainstem (GTV = PTV)
<1 cm^3^	1 × 24 Gy	1× 16Gy
1–10 cm^3^	1 × 21 Gy	1 x 16Gy
10–20 cm^3^	1 × 18 Gy	1 x 16Gy
20–65 cm^3^	1 × 15 Gy	No SRS

All BMs are dosed equally in the same patient. If the V12 Gy exceeds 10 cm^3^ of the healthy brain tissue nearby the largest brain metastasis, it is allowed to lower the fraction dose to a single dose of 21, 18 or 15 Gy

Patients will be immobilised in a supine position within a thermoplastic mask or stereotactic noninvasive frame, with or without bite block and/or other fixation, according to institutional standards for SRS. The accuracy of the stereotactic fixation system should be good enough to justify the CTV to PTV margin used. This means the intrafraction motion should at least be within the CTV-PTV margin used. If a margin of 0 mm is used, the maximum intrafraction motion should be <0.5 mm, with the SD being less than 0.25 mm. A planning CT scan with ≤2 mm thick contiguous slices (preferable CT slice thickness = 1 mm) will be fused to a contrast-enhanced stereotactic MRI scan. The interval between the planning-MRI and actual SRS treatment is a maximum of 3 weeks. Single or multiple isocentres are allowed for delivering SRS according to the preference of treatment center. Tissue density inhomogeneity correction will be used. Positional verification and correction prior to (and/or during) radiation should be executed according to the institutional protocol for stereotactic radiotherapy and should be in accordance with the CTV-PTV margin used. All techniques that result in the dose requirements being met are allowed. Participating institutes will have to define their radiation delivery treatment prior to the initiation of the study. Techniques that have a shorter treatment time duration are preferred as this is more comfortable for the patient and might prevent an increase in the intrafraction displacement over the treatment time. All vendors are allowed to deliver SRS, such as a linear accelerator, Gamma Knife and CyberKnife.

### Number of patients and recruitment

Questionnaires measuring QOL with the EQ5D EUROQOL questionnaire are collected from patients with multiple BM treated with WBRT or SRS at baseline and at 3 months after treatment. For this phase III trial, sample size calculation is based on the clinically relevant difference of 0.10 points of the EQ5D-5 L index value (range 0–1) at 3 months after treatment with regard to baseline, with a standard deviation of 0.25 points. For every patient this difference in EQ5D-5 L QOL score is calculated (score at 3 months minus score at baseline). The average score of all patients in the SRS group is calculated and this average score is compared to the calculated average score of all patients in the WBRT group. This is accordingly the method described by Pickard. [6] Sample size calculation is performed for a comparison of means with two-sided alpha 0.05 and power of 0.80. This leads to a sample size per group of 100 patients. To account for drop out, the sample size for this study will be increased by 15% to 230 patients (115 per group). After 86 patients treated, an interim analysis is performed to monitor the safety of the trial regarding the experimental SRS arm.

Patient accrual was started on 1^st^ July, 2016. Up to 14^th^ April, 2017, nine patients were randomised. With expected participation of 12 centres, it is estimated that patient’s accrual can be completed within 2 years with an expected accrual of 10–15 patients each year per centre.

For this phase III study, a comparison of the above described difference in EQ5D score between SRS and WBRT group will be performed using an independent samples Student’s t-test with two-sided significance level alpha set at 0.05. A clinical significant difference in EQ5D is determined at 0.10 points in the index score. [4] For the total patient cohort, a multivariate analysis is performed to identify prognostic factors for a difference in EQ5D score at 3 months with regard to baseline. Differences in secondary endpoints that repeat in time are analysed with Kaplan Meyer curves including log-rank test or ANOVA test. Time-to-event data (e.g. overall survival) will be compared using Kaplan–Meier curves and log-rank test. Means will be compared using independent samples Student’s t-tests. Frequencies (e.g. WHO-PFS) will be compared using Chi-square test.

## Discussion

A typical SRS treatment requires a high-resolution contrast-enhanced planning-MRI, frameless mask, a planning-CT, a treatment plan and quality assurance. Generally, all preparations will take a maximum of 2 weeks. Duration of SRS delivery depends on the number of isocentres, arcs and dose rate, but usually in the range of several minutes up to 45 min. With WBRT, all other preparation steps are required, but treatment planning and quality assurance are less complex. WBRT treatment is delivered in five fractions compared to a single treatment fraction with SRS. Questionnaires will be assessed at baseline, 3 months after treatment and every 3 months thereafter until 1 year after treatment. Questionnaires can be assessed by telephone, or during scheduled outpatient clinic visits. Not all hospitals have the logistic capacity to perform neurocognitive tests and extensive QOL assessment; hence, neurocognition (Hopkins Verbal Learning Test) and more extensive QOL (EORTC QLQ-C30, EORTC BN20, and EORTC QLQ-FA13) will be facultative endpoints and will only be performed at baseline and at 3 months after radiotherapy at the outpatient clinic in centres willing to participate. Although these secondary endpoints are facultative, these endpoints will also provide valuable information, and participating centres are recommended to monitor them.

## Abbreviations

BM: brain metastases
CT: computed tomography
CTCAE: common criteria of adverse events
GTV: gross tumour volume
Gy: Gray
ICRU: International Commission on Radiation Units & Measurements
MRI: magnetic resonance imaging
PTV: planning target volume
QOL: quality of life
SRS: stereotactic radiosurgery
WBRT: whole brain radiotherapy
